# Recent Advances in Multiple Strategies for the Biosynthesis of Sesquiterpenols

**DOI:** 10.3390/biom15050664

**Published:** 2025-05-03

**Authors:** Mengyuan Li, Ruiqi Chen, Jianjun Qiao, Weiguo Li, Hongji Zhu

**Affiliations:** 1 Department of Pharmaceutical Engineering, School of Chemical Engineering and Technology, Tianjin University, Tianjin 300072, China; lmy3276@tju.edu.cn (M.L.); ruiqichen@tju.edu.cn (R.C.); jianjunq@tju.edu.cn (J.Q.); 2 State Key Laboratory of Synthetic Biology, Tianjin University, Tianjin 300072, China; 3 Zhejiang Institute of Tianjin University (Shaoxing), Shaoxing 312300, China

**Keywords:** sesquiterpenols, bioactivities, metabolic engineering, enzyme engineering, synthetic biology

## Abstract

Sesquiterpenols, a class of natural compounds composed of three isoprene units that form a 15-carbon skeleton with hydroxyl (-OH) group, are characterized by their volatility and potent aromatic properties. These compounds exhibit a wide range of biological activities, including antitumor, antibacterial, anti-inflammatory, anti-neurotoxic, antiviral, immunosuppressive, hepatoprotective, and cardiotonic effects. Due to their diverse physiological functionalities, sesquiterpenols serve as critical raw materials in the pharmaceutical, food, and cosmetic industries. In recent years, research on the heterologous synthesis of sesquiterpenol compounds using microbial systems has surged, attracting significant scientific interest. However, challenges such as low yields and high production costs have impeded their industrial-scale application. The rapid development of synthetic biology has introduced innovative methodologies for the microbial production of sesquiterpenol compounds. Herein, we examine the latest synthetic biology strategies and progress in microbial sesquiterpenol production, focusing on adaptive sesquiterpenol synthase screening and expression, synthesis pathway regulation, intracellular compartmentalized expression strategies, and tolerance to terpenoid-related toxicity. Critical challenges and future directions are also discussed to advance research in sesquiterpenol biosynthesis.

## 1. Introduction

Sesquiterpenols, a class of terpenoids with C15-carbon skeletons, are structurally classified into acyclic, monocyclic, bicyclic, tricyclic, and tetracyclic derivatives based on their carbon ring numbers [1]. These natural products are widely found in plants, fungi, bacteria, and insects with a strong fragrance that make them widely used in the flavor and perfume industries. Beyond their fragrance applications, numerous sesquiterpenols have anticancer [1], antimicrobial [2], anti-inflammatory [3], and other pharmacological activities. Their structural versatility also positions them as promising candidates for advanced biofuels or biofuel precursors. So, they have broad research value and commercial applications in cosmetics, food, pharmaceuticals, agriculture, and biofuels. Notable examples include α-bisabolol and nerolidol, which serve as drug precursors [4,5]; while the simplest linear sesquiterpenol, farnesol, has been studied for its potential as jet fuel [6].

Sesquiterpenols, despite their significant bioactivities and broad application value, are mainly produced through plant extraction and chemical synthesis [7]. However, both methods have notable limitations. Plant extraction is hindered by limited and unstable natural resources, environmental sensitivity (e.g., seasonal, climatic, and soil factors), and relatively low extraction efficiency [8]. Chemical synthesis typically requires energy-intensive conditions, such as high-temperature and high-pressure operations, as well as specialized equipment. These processes often yield products of low purity, generate numerous intermediate by-products, and ultimately result in low final yields, primarily due to the inherent structural complexity and chiral isomer diversity of sesquiterpenols [9].

With the development of metabolic engineering and synthetic biology, many sesquiterpenols have been successfully produced in engineered microorganisms. Microbial cell factories, engineered through synthetic biology, offer a promising alternative to conventional plant extraction and chemical synthesis. They effectively circumvent the inherent economic and environmental limitations of these conventional methods, while facilitating efficient biomass conversion and promoting sustainable resource utilization. Synthetic biology provides an innovative approach for the sustainable production of sesquiterpenols using microbial hosts, such as *Saccharomyces cerevisiae* and *Escherichia coli*. Yeast has gradually developed as the main platform for sesquiterpenol biosynthesis due to its superior compatibility with plant-derived terpenoid biosynthetic enzymes and precision genome editing systems. Current engineering includes examples through integrating directed evolution and protein engineering to optimize rate-limiting enzymes, which enable the construction of robust microbial platforms capable of producing structurally diverse sesquiterpenols. Furthermore, systematic process optimization and metabolic network refinement further enhance production indices, including titer and yield, which are critical parameters for industrial-scale production.

In this review, we summarize recent advances in the microbial synthesis of sesquiterpenols, including critical biosynthetic pathways, engineered strategies, and emerging application prospects. In addition, we discuss the challenges in the field, aiming to provide new insights and strategies for the sustainable production of sesquiterpenols for future research.

## 2. Sesquiterpenols and Their Bioactivities

**Figure 1:**
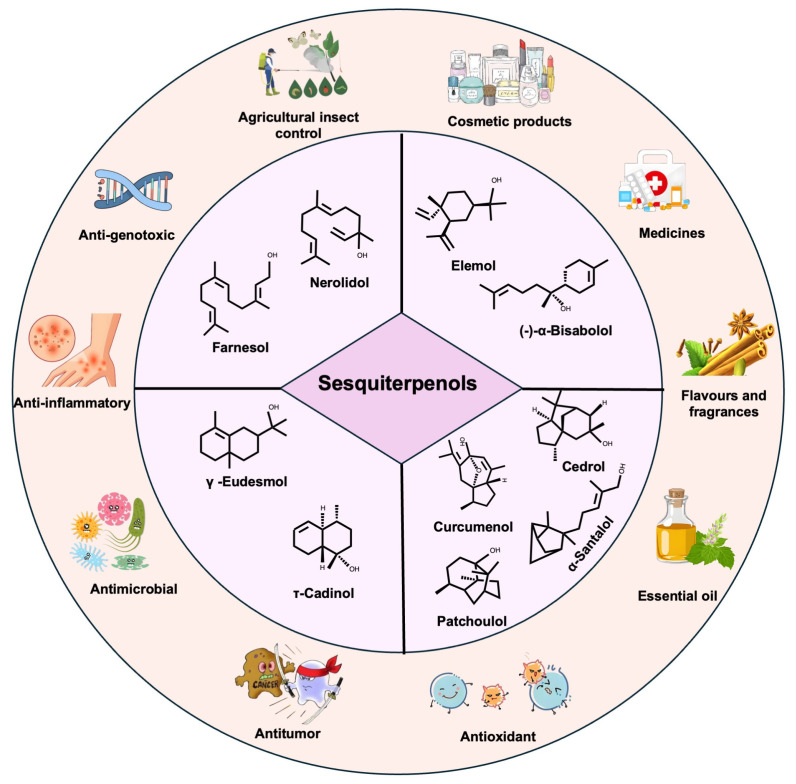
Applications, biological activities and structures of sesquiterpenols.

Sesquiterpenols can be structurally classified based on the number of carbocyclic rings in their molecular framework (Figure 1), including acyclic (linear), monocyclic, bicyclic, and polycyclic subtypes.

### 2.1. Acyclic Sesquiterpenols

Acyclic sesquiterpenols are defined as open chain sesquiterpenol compounds, with representative examples including nerolidol and farnesol (Table 1, Figure 1). Nerolidol exists as a colorless to pale yellow viscous oil, and is characterized by its delicate sweet floral aroma, which includes nuances of rose, lily-of-the-valley, and apple blossom [10]. This compound is primarily derived from plant sources, such as *Melaleuca viridiflora*, *Zingiber officinale, Myroxylon balsamum*, and *Myroxylon peruiferum*, and trace quantities can be detected in premium *Roman chamomile* essential oils [10]. Nerolidol exhibits diverse bioactivities, including antimicrobial [11], anti-biofilm [12], antioxidant [13], antiparasitic [14], transdermal permeation-enhancing [15], antinociceptive [16], and anticancer [17] properties. Farnesol, a colorless hydrophobic liquid with limited aqueous solubility but high miscibility in organic solvents, such as ethanol, is renowned for its sweet floral fragrance. It is ubiquitously distributed in essential oils of diverse botanical species, notably *Aniba rosaeodora*, *Abelmoschus moschatus*, *Cananga odorata*, *Acacia farnesiana*, *Myroxylon balsamum*, *Cymbopogon martinii*, *Polianthes tuberosa*, *Jasminum grandiflorum*, and *Citrus aurantium* [18]. In addition to plant systems, farnesol is present in animal and microbial organisms, where it plays crucial roles in intercellular signaling, quorum sensing modulation, and apoptosis regulation [19]. These distinctive characteristics support its extensive applications as a cosmetic fragrance component, a natural acaricidal agent, and a precursor in anticancer drug development [19].

### 2.2. Monocyclic Sesquiterpenols

Monocyclic sesquiterpenols are defined as sesquiterpenols containing a single carbocyclic structure, with representative examples including (-)-α-bisabolol and elemol (Table 1, Figure 1). The compound (-)-α-Bisabolol exists as a colorless to straw-yellow viscous liquid predominantly found in essential oils of *Chamomilla recutita*, *Populus balsamifera*, and some species within the *Myoporum* and *Salvia* genera [36]. This compound exhibits biological activities, such as ulcer healing [20], analgesia [9], anti-inflammatory effects [37], bacteriostasis [20], anti-genotoxic properties [20], and melanin inhibition [20]. Its pronounced anti-inflammatory and skin-soothing properties have enabled its widespread pharmaceutical and cosmeceutical applications [38]. Elemol, identified in *Amomum tsao-ko* [39], *Cymbopogon winterianus*, and traditional Chinese medicinal materials, such as *Illicium verum* and *Eriobotrya japonica* [21], is valued for its distinctive aromatic profile and chemical stability. These attributes make it a suitable fixative agent or flavor enhancer in fragrance formulation, personal care products, laundry detergents, and household commodities [21]. Additionally, elemol serves critical phytochemical defense functions against the pathogenic microorganisms in plants [40].

### 2.3. Bicyclic Sesquiterpenols

Bicyclic sesquiterpenols are defined as sesquiterpenols containing two carbocyclic structures, with representative examples including eudesmol and τ-cadinol (Table 1, Figure 1). Eudesmol is found in natural essential oils, and has many isomers, such as α-, β-, and γ-eudesmol [40]. These isomers are typically a colorless to pale yellow viscous liquid with camphoraceous aroma [40]. Due to its antimicrobial and insecticidal properties, it can be used as a preservative which is widely applied in pharmaceuticals and the food industry [22]. Additionally, eudesmol can serve as a fragrance fixative and a bergamot oil substitute via its acetate derivative. Tau-cadinol is an aromatic bicyclic sesquiterpenol, which is primarily isolated from the fresh root bark of *Zanthoxylum piasezkii*, and demonstrates exceptional pest-repellent efficacy during herbal medicine storage, particularly against *Tribolium castaneum* and *Lasioderma serricorne* [41]. This compound is distinguished by its operational efficiency, minimal residue profile, cost-effectiveness, and environmental compatibility [41]. Beyond its agricultural applications [41], τ-cadinol exhibits the multifaceted potential as a nutritional substrate [42], membrane stabilizer [43], and energy metabolism modulator [43]. Its industrial applications extend to surfactant formulations and emulsifier production. Pharmacological investigations reveal additional bioactivities, including smooth muscle relaxation [23], antidiarrheal effects [25], calcium channel antagonism [24], phyto germination regulation [26], acaricidal action, and insect repellency [26].

### 2.4. Polycyclic Sesquiterpenols

Polycyclic sesquiterpenols encompass compounds with three or more carbocyclic structures, with representative examples including patchoulol, α-santalol, cedrol, and curcumol (Table 1, Figure 1). Patchoulol, a tricyclic sesquiterpenol, constitutes the principal component of patchouli essential oil. It exists as a white to off-white solid under ambient conditions. This compound is primarily derived from *Pogostemon cablin*, where its essential oil is obtained through steam distillation of the leaves, stems, and roots [44]. Beyond its characteristic persistent woody aroma, which facilitates its extensive use in cosmetics and household products, patchoulol demonstrates therapeutic potential in Alzheimer’s disease prevention, anti-anginal activity [27], arrhythmia suppression [27], and antihypertensive effects [27]. Alpha-Santalol, a tricyclic sesquiterpenol primarily derived from the essential oils of *Santalum album* [45], has been shown to possess broad-spectrum antimicrobial properties. It effectively inhibits the growth of several notable pathogens [29], including *Helicobacter pylori*, *Candida albicans*, and *Escherichia coli*. Given its multifunctional bioactivities, including antibacterial prophylaxis [29], virucidal activity [31], antioxidant properties [30], and antitumor potential [28], α-santalol is positioned as a promising pharmaceutical candidate. Cedrol, a tricyclic sesquiterpenol presenting as white crystals with a mild cedarwood aroma, finds extensive application in woody and oriental fragrance formulations, particularly in soap and detergent perfumery [46]. Pharmacological studies reveal its anti-inflammatory [33], analgesic [33], cardioprotective [32], antitumor [34], hair growth-promoting, and antimalarial properties [46]. Curcumol, an orange-yellow crystalline tricyclic sesquiterpenol isolated from *Curcuma longa* [35], is a bitter-tasting compound that demonstrates a wide range of bioactivities, including anticancer, antimicrobial, antifungal, antiviral, and anti-inflammatory effects [47].

## 3. Sesquiterpenol Biosynthesis

### 3.1. Biosynthetic Pathway

**Figure 2:**
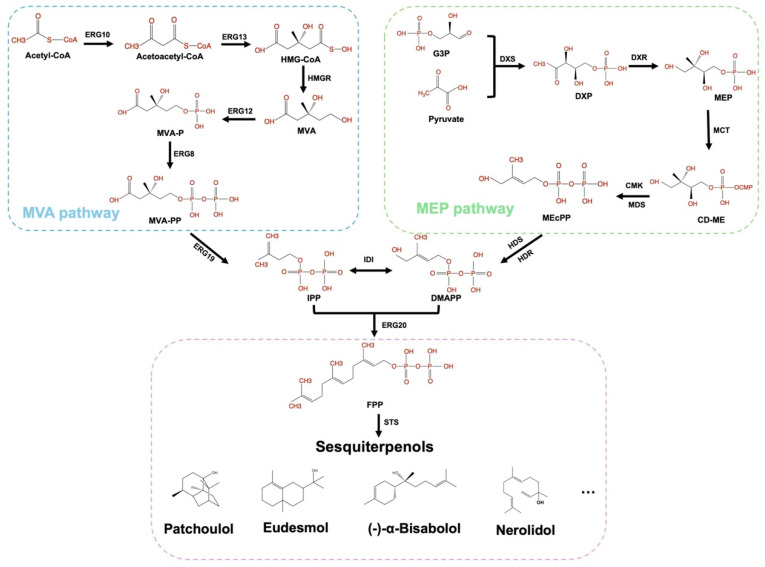
Metabolic pathway of sesquiterpenols biosynthesis. Abbreviation of metabolites: HMG-CoA, 3-hydroxy-3-methylglutaryl-CoA; MVA, mevalonate; MVA-P, 5-phosphomevalonate; MVA-PP, 5-diphosphomevalonate; IPP, isoprene diphosphate; DMAPP, dimethylallyl diphosphate; FPP, farnesyl diphosphate; G3P, glyceraldehyde-3-phosphate; DXP, 1-deoxy-D-xylulose-5-phosphate; MEP, 2-C-Methyl-d-erythritol-4-phosphate; CD-ME, 4-(cytidine-5′-diphospho)-2-C-methyl-D-erythritol; ME-cPP, 2-C-methyl-D-erythritol-2,4-cyclodiphosphate; ERG10, acetoacetyl-CoA thiolase; ERG13, 3-hydroxy-3-methylglutaryl-CoA synthase; HMGR, 3-hydroxy-3-methylglutaryl-CoA reductase; ERG12, mevalonate kinase; ERG8, phosphomevalonate kinase; ERG19, mevalonate diphosphate decarboxylase; IDI, isopentenyl diphosphate isomerase; DXS, 1-deoxy-D-xylulose-5-phosphate synthase; MCT, 2-C-methyl-D-erythritol-4-phosphate cytidylyltransferase; CMK, 4-diphosphocytidyl-2-C-methyl-D-erythritol kinase; MDS, 2-C-methyl-D-erythritol-2,4-cyclodiphosphate synthase; HDS, 4-hydroxy-3-methylbut-2-enyl-diphosphate synthase; HDR, 4-hydroxy-3-methylbut-2-enyl diphosphate reductase; ERG20, geranyl/farnesyl diphosphate synthase; STS, sesquiterpenol synthase.

Farnesyl pyrophosphate (FPP) serves as the direct biosynthetic precursor for sesquiterpenol synthases in the production of sesquiterpenols. Isopentenyl pyrophosphate (IPP) and dimethylallyl pyrophosphate (DMAPP), the universal precursors for FPP biosynthesis, also constitute the fundamental building blocks for diverse terpenoid compounds [48]. These isoprenoid precursors are naturally derived from two distinct metabolic routes: the mevalonate (MVA) pathway and the methylerythritol phosphate (MEP) pathway, as shown in Figure 2 [49]. The MEP pathway exists in bacteria, cyanobacteria, green algae, and plant plastids; while the MVA pathway is found in eukaryotic microorganisms, archaea, and the cytoplasm of some higher plants [50]. In the chassis *Escherichia coli*, terpenoid precursor supply is exclusively mediated through the MEP pathway, which is initiated with glyceraldehyde 3-phosphate (G3P) and pyruvate as substrates. The catalytic activities of 1-deoxy-D-xylulose-5-phosphate synthase (DXS) and 1-deoxy-D-xylulose-5-phosphate reductoisomerase (DXR) represent the rate-limiting steps in this pathway [51,52]. In yeast systems, including *Saccharomyces cerevisiae* and *Yarrowia lipolytica*, the MVA pathway is used for sesquiterpenol precursor biosynthesis. Acetyl-CoA serves as the precursor for this pathway, with the rate-limiting step is the reduction of 3-hydroxy-3-methylglutaryl-CoA (HMG-CoA) to mevalonate by HMG-CoA reductase [51,52].

**Figure 3:**
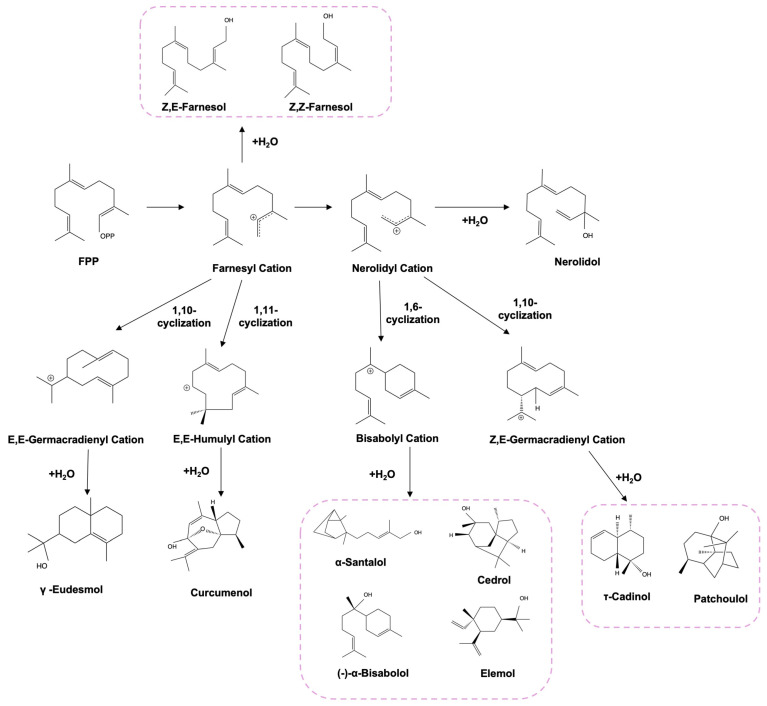
Reaction mechanism of STSs. (E,E)-Germacradienyl cations (C1-C10 closure) and (E,E)-humulyl cations (C1–C11 closure) are initiated from FPP. The neryl diphosphate (NPP), isomerized from FPP, can be cyclized into bisabolyl cations (C1–C6 closure) and (Z,E)-germacradienyl cations (C1–C10 closure). Both farnesyl cations and nerolidyl cations have the potential to directly undergo deprotonation or hydration, resulting in the formation of acyclic sesquiterpenols, such as farnesenol and nerolidol.

Under the catalysis of farnesyl diphosphate synthase (ERG20), geranyl diphosphate (GPP, the electrophilic partner) and isopentenyl diphosphate (IPP, the nucleophilic partner) generated farnesyl diphosphate (FPP), which serves as the critical precursor. The subsequent transformation of FPP by sesquiterpene synthases yields structurally diverse sesquiterpenoids [53]. Sesquiterpenols, as a prominent subclass of these compounds, exhibit remarkable structural diversity driven by varied cyclization patterns arranged by STS catalysis. The enzymatic process is initiated by FPP-derived carbocation formation, generating (E, E)-germacradienyl cations (C1–C10 closure) and (E, E)-humulyl cations (C1-C11 closure). Alternatively, FPP isomerization produces neryl diphosphate (NPP), which undergoes cyclization to form distinct carbocation intermediates, including bisabolyl cations (C1–C6 closure) and (Z,E)-germacryl cations (C1–C10 closure) [54]. These intermediates undergo further deprotonation or hydration, ultimately yielding cyclic sesquiterpenols. Notably, the farnesyl cation and nerolidyl cation demonstrate alternative stabilization pathways through undergoing deprotonation or hydration, giving rise to acyclic sesquiterpenols, such as farnesol [55] and nerolidol [56] (Figure 3).

### 3.2. Sesquiterpenol Synthases

Sesquiterpene synthases catalyze the conversion of farnesyl diphosphate (FPP) into unsaturated hydrocarbon scaffolds, including farnesene and bisabolene, through Mg^2^⁺-dependent pyrophosphate elimination. By contrast, sesquiterpenol synthases utilize the same FPP substrate to generate oxygenated derivatives via distinct hydroxylation mechanisms [53]. Both enzyme classes operate through a conserved catalytic framework. First, the metal-coordinated cleavage of FPP generates a reactive farnesyl carbocation intermediate. Then, carbocation rearrangement via hydride shifts, cyclization, or ring modifications. Finally, termination is achieved through deprotonation to yield hydrocarbons in sesquiterpene synthases or via hydroxylation to produce sesquiterpenol in sesquiterpenol synthases [57]. Hydroxylation of the sesquiterpenol synthases proceed through two distinct pathways. The direct hydration mechanism employs strategically positioned hydrophilic residues (Thr/Ser) in the active sites of sesquiterpenol synthases to the coordinated water-mediated protonation of the carbocation intermediates, as exemplified in patchoulol biosynthesis [58]. Alternatively, oxidative post-modification involves sequential actions of sesquiterpenol synthases and cytochrome P450 monooxygenases (CYP450s), where neutral hydrocarbon products from sesquiterpenol synthases undergo subsequent hydroxylation, as observed in santalol biosynthesis [59]. Structurally, both sesquiterpene synthases and sesquiterpenol synthases belong to the terpene synthase (TPS) superfamily. They are characterized by conserved α-domain architecture and Mg^2^⁺-binding motifs (DDxxD, NSE/DTE). Sesquiterpene synthases have evolved precise active-site topographies that enable stereochemical control over single hydrocarbon products [57]. Phylogenetic evidence supports the emergence of sesquiterpenol synthases from sesquiterpene synthase ancestors through adaptive mutations that introduced hydrophilic residues, facilitating water access [46].

## 4. Common Strategies for Optimizing Sesquiterpenol Biosynthesis

To date, six sesquiterpenols have been employed in biosynthetic processes. These include acyclic sesquiterpenols, such as farnesol and nerolidol, monocyclic sesquiterpenols, like bisabolol, bicyclic sesquiterpenols, such as cedrol, and polycyclic sesquiterpenols, including patchoulol and santalol. Given the significant potential of biosynthetic pathways for sesquiterpenols production, numerous effective strategies have been developed to optimize biosynthetic production (Table 2). This review focuses on recent advances and examines the optimization approaches for sesquiterpenol biosynthesis from four key perspectives: enzyme engineering, metabolic regulation, subcellular organelle engineering, and tolerance to terpenoid-related toxicity.

### 4.1. Enzyme Engineering

#### 4.1.1. Identification and Functional Verification of Key Enzymes

**Figure 4:**
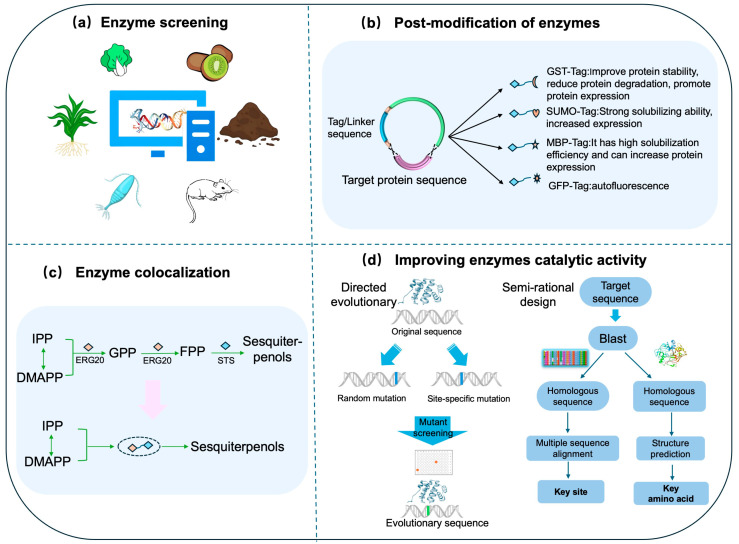
Enzyme engineering for improving sesquiterpenol production: (**a**) enzyme screening; (**b**) post-modification of enzymes; (**c**) enzyme colocalization; (**d**) improving enzymes catalytic activity.

The biosynthesis and utilization of farnesyl pyrophosphate (FPP) are pivotal factors that directly influence the final titers of sesquiterpenol production. The rapid advancement of DNA sequencing technologies has made genetic sequence information available in public databases. This enables the identification of potential gene sequences for heterologous expression in microbial chassis, which can achieve high-yield production (Figure 4a, Table 2). The crucial genes identified thus far that are involved in sesquiterpenol biosynthesis are listed in Table 3. To date, nerolidol synthases (NES) have been identified from five distinct plant species [65,83,84,85,86], with functional validation achieved in *Saccharomyces cerevisiae* for enzymes derived from *Actinidia chinensis* and *Celastrus angulatus*. Green et al. pioneered the identification of the nerolidol synthase AcNES1 from *A. chinensis* flowers [83], while Li et al. characterized two NES isoforms (CaNES1 and CaNES2) from *C. angulatus*. Subsequent metabolic engineering strategies integrating these genes into *S. cerevisiae* resulted in engineered strains with enhanced nerolidol yield [65]. Notably, significant interspecies variability in enzymatic properties has been observed. The AcNES1 gene from *A. chinensis* is currently the most widely used variant in research. For α-bisabolol biosynthesis, functional characterizations of α-bisabolol synthases have been conducted in several plants via transcriptomic and genomic analyses, including *Matricaria recutita* [87], *Lippia dulcis* [88], *Sesamum indicum* [89], and *Arabidopsis thaliana* [90]. Tai et al. elucidated the biosynthesis and regulation of α-bisabolol in *M. recutita*, by identifying FPP synthase (MrFPS), α-bisabolol synthase (MrBBS), and related transcription factors of the WRKY, AP2/ERF, and MYB families [87]. These studies collectively provide critical insights into the transcriptional regulation of sesquiterpenol biosynthesis and establish a working model for finding new enzymes and improving biosynthetic pathways in sesquiterpenol production, which uses multi-omics data and synthetic biology tools.

#### 4.1.2. Post-Modification of Enzymes

Strategic fusion of appropriate tags has been shown to enhance protein stability and augment recombinant expression efficiency (Figure 4b, Table 2) [98]. This approach offers a versatile tool for optimizing enzyme stability, solubility and expression levels. Unlike large fusion tags that may cause steric hindrance, short peptide tags preserve the native protein structure and enhance translational initiation efficiency, thereby preserving enzymatic functionality [99]. Commonly used short tags include thioredoxin A (TrxA), maltose-binding protein (MBP), glutathione S-transferase (GST), small ubiquitin-like modifier (SUMO), N-utilizing substance A (NusA), and various short peptide tags [100]. The T7A tag, a 22-amino-acid peptide with strong acidic properties, is an example of this strategy. It greatly reduces inclusion body formation and enhances soluble expression in *E. coli*. For instance, for patchoulol synthase (PTS2), C-terminal fusion with the T7A tag reduced the inclusion body propensity by 39.74% and boosted patchoulol yield simultaneously [74]. Complementary enhancement also can provide through chaperone co-expression. In *E. coli*, the DnaK/DnaJ chaperone system, a member of the Hsp70 family, improves recombinant protein solubility through multiple mechanisms, including facilitating folding, preventing aggregation, and disaggregating proteins. Lu et al. reported a 10% increase in α-bisabolol yield by co-expressing DnaK and DnaJ [5]. These findings collectively demonstrate short peptide tags as powerful tools for optimizing soluble expression of challenging heterologous proteins in *E. coli*, particularly when combined with molecular chaperone systems. The T7A paradigm shows that a rationally designed tag can overcome expression bottlenecks while preserving catalytic activity, making this approach widely applicable in microbial metabolic engineering.

#### 4.1.3. Enzyme Colocalization

Enzyme colocalization represents a pivotal strategy for enhancing metabolic efficiency and product yield in biosynthetic pathways (Figure 4c, Table 2). This approach fuses key enzymes into multi-enzyme complexes, increasing local concentrations of pathway enzymes and metabolites while establishing substrate channeling [101]. In sesquiterpenol biosynthesis, enzyme fusion strategies have proven effective for metabolic engineering, with farnesyl pyrophosphate (FPP) serving as the critical precursor [102]. The inherent metabolic competition for FPP and product feedback inhibition often limits sesquiterpenol production. Recent advances in constructing fusion proteins of FPP synthase (FPPS) with sesquiterpenol synthases have demonstrated remarkable improvements in yield. Previous studies have shown that the composition and length of peptide linkers in enzyme fusions can affect terpene production. Consequently, Cheah et al. designed FPPS–NES fusion constructs incorporating four distinct peptide linkers. Each of these four fusion constructs achieved a remarkable >110-fold increase in nerolidol production, yielding titers between 3.51 and 4.20 g/L in culture [56]. Luo et al. found that fusing PTS to the C-terminus of ERG20, rather than the N-terminus, greatly increased patchoulol yield [80]. To understand why, they generated protein 3D models using the AlphaFold2 Multimer. The simulations revealed that the active-site distance between ERG20 and PTS was shorter in the ERG20–PTS fusion than in the PTS–ERG20 fusion. Similarly, Ma et al. [44] and Zhou et al. [74] also utilized the ERG20–PTS fusion protein for patchoulol synthesis. These studies together prove that the fusion–protein strategy has been used in various microbial chassis with significant outcomes. Biomolecular scaffolding systems also facilitate enzyme colocalization. Scaffold-mediated enzyme organization, which can be categorized into protein-based and nucleic acid-based systems, offers an alternative approach to spatial control [103]. Tippmann et al. engineered an affibody scaffold in *S. cerevisiae* to colocalize FPPS and farnesene synthase, increasing farnesene yield by 135% under glucose-fed conditions [104]. Additionally, Han et al. utilized Tya, a component of the Ty1 retrotransposon, to assemble VLPs by incorporating three farnesol biosynthetic enzymes in *S. cerevisiae*, thereby quadrupling farnesol production [99].

These colocalization strategies synergistically optimize enzyme proximity, substrate channeling, and metabolic flux partitioning. The fusion protein approach is particularly effective in redirecting FPP flux toward target sesquiterpenols. Scaffold systems enable modular control of enzyme stoichiometry. The success of these strategies across various microbial chassis underscores their universal applicability in sesquiterpenol biosynthesis, and provides crucial technical support for industrial-scale production. Further breakthroughs in metabolic pathway optimization are anticipated as programmable scaffolding systems are developed and AI-driven fusion protein design advances.

#### 4.1.4. Improving Enzymes Catalytic Activity

Site-directed mutagenesis has emerged as a principal methodology for enzyme optimization in sesquiterpenol biosynthesis, enabling enhanced catalytic efficiency and product yields (Figure 4d, Table 2). As most sesquiterpenol synthases originate from plants and are poorly expressed in microbial systems, strategic mutagenesis of these enzymes is crucial for enhancing production efficiency at the industrial scale. Homologous sequence alignment aids in identifying functional residues that dictate enzyme activity, stability, and substrate specificity. This approach has proven effective in numerous studies. For example, Zhou et al. substituted specific amino acid residues in PTS3 to more conserved ones, identified through a comparative analysis of homologous proteins. This resulted in a hybrid mutant, PTS3mut4, which enhanced patchoulol production by 2.02-fold [42]. Similarly, Lei et al. identified non-conserved residues C41 and Y85 in Mvan4662 through multi-sequence alignment. Introducing the C41S/Y85F mutations, along with overexpression of the phosphatase NudJ, which enabled *E. coli* to produce 572.13 mg/L of Z,Z-farnesol [6]. Homology modeling predicts 3D structures, enabling rational enzyme design and expanding the range of possible products. Srivastava et al. employed simulation-guided engineering to create PTS variants that eliminate water capture. They also demonstrated that modifying the structurally conserved Hα-1 loop reduces hydroxylation in PTS, thereby producing cyclic neutral intermediates. This Hα-1 loop modification could serve as a general strategy for engineering sesquiterpenol synthases to produce complex cyclic hydrocarbons [105]. Homology modeling can also boost catalytic efficiency. Liu et al. optimized nerolidol synthase FaNES1 via homology modeling, creating the G498Q mutant, which increased trans-nerolidol yield 3.16-fold to 379 mg/L [66]. Similarly, Cheng et al. used the plmDCA algorithm to design α-bisabolol synthase BOS mutants. The F324Y variant showed a 73% yield improvement [106].

These mutagenesis strategies show three key innovation paradigms: residue-targeted precision engineering (conserved motif modification), structure-informed loop remodeling (active site optimization), and algorithm-driven combinatorial mutagenesis (machine learning-guided design). The proven enhancements establish protein engineering as a cornerstone for industrial sesquiterpenol production. Future developments integrating deep mutational scanning with AI-predictive models promise unprecedented enzyme optimization.

### 4.2. The Strategies for the Regulation of the Synthesis Pathway

#### 4.2.1. The Engineering of Host Metabolism

**Figure 5:**
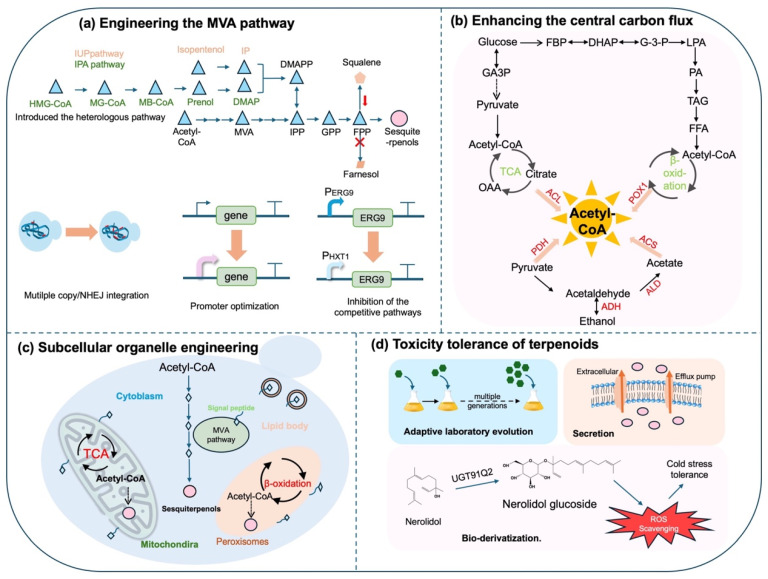
The regulation of the synthesis pathway, subcellular compartmentalization, and toxicity tolerance engineering: (**a**) engineering the MVA pathway; (**b**) enhancing the central carbon flux; (**c**) subcellular compartmentalization; (**d**) strategies of improving cell toxicity tolerance in terpenoid production: secretion; adaptive laboratory evolution (ALE); bio-derivatization.

The production of sesquiterpenols via microbial endogenous metabolism alone remains insufficient for industrial applications. A common strategy to enhance their production involves engineering rate-limiting pathways to improve precursor supply (Figure 5a, Table 2). The HMG1 protein, a reductase that catalyzes the conversion of HMG-CoA to mevalonate, is the rate-limiting enzyme in the MVA pathway and represents a critical bottleneck in terpenoid biosynthesis. Overexpression of HMG1 significantly enhances metabolic flux through the mevalonate pathway. During farnesol (FOH) biosynthesis, ten genes in the MVA pathway were overexpressed to assess their contributions to FOH production. Among these, overexpression of HMG-CoA reductase (HMG1) demonstrated the most pronounced enhancement [61]. In *S. cerevisiae*, HMG1 activity is regulated by feedback and cross-regulation in the MVA pathway, primarily through protein degradation via its SSD domain [107]. Truncating the SSD domain prevents post-transcriptional degradation of HMG1, thereby enhancing its catalytic stability [108]. Consequently, the N-terminal truncated tHMG1 (500 amino acids) exhibits stable cytoplasmic expression. By overexpressing this truncated HMG-CoA reductase (tHMGR1), Guo et al. increased farnesol production 50.8-fold to 5.08 mg/L [55]. Given the multi-enzyme nature of the MVA pathway, combinatorial overexpression strategies generally outperform single-enzyme optimization. Ma et al. overexpressed tHMG1, and the resulting strain showed a 2.5-fold increase of the (-)-α-bisabolol yield. When both tHMG1 and ERG20 were overexpressed, the final production of (-)-α-bisabolol was increased 3.4-fold compared with the initial engineered strain. Thus, it can be concluded that overexpression of tHMG1 and ERG20 can effectively increase the FPP supply, thereby significantly enhancing the production of (-)-α-bisabolol [70]. Recent advances include the Global Metabolic Engineering Strategy (GMES), which employs cocktail δ-integration in *S. cerevisiae* to enable multi-copy integration of pathway genes at δ-sites for complex pathway optimization [109]. Ryosuke Mitsui et al. applied this strategy and achieved patchoulol production at 42.1 mg/L through pathway gene copy number optimization [75].

Introducing heterologous pathways is a commonly used strategy to enhance precursor availability (Figure 5a, Table 2). Recent advances in heterologous terpenoid biosynthesis have gradually been increasingly reported and applied. Lu et al. successfully integrated the heterologous MVA pathway into *S. marcescens* and used inducible and constitutive promoters to adjust key enzyme expression levels, thereby increasing (-)-α-bisabolol yield to 2.1 g/L [5]. Seon-Won Kim et al. successfully expressed the mevalonate pathway in *E. coli*, achieving a farnesol concentration of 526.1 mg/L [110]. The isopentenol utilization pathway (IUP), a two-step enzymatic cascade, enables precursor supplementation via exogenous isopentenol administration [111]. This system utilizes *Arabidopsis thaliana* choline kinase (CK) and isopentenyl phosphate kinase (AtIPK) to sequentially phosphorylate isopentenol, thereby generating isopentenyl diphosphate (IPP) and dimethylallyl diphosphate (DMAPP)—universal precursors for terpenoid biosynthesis. Ma et al. introduced the IUP pathway in *S. cerevisiae*, achieving a remarkable 147-fold increase in the IPP/DMAPP precursor pool compared to native MVA pathway [112]. Furthermore, Clomburg et al. developed the isoprenoid alcohol (IPA) pathway [113], which demonstrates superior energetic efficiency through reduced ATP and cofactor demands, thereby establishing a versatile platform for isoprenoid production. Overexpression of IPP isomerase (IDI) is also a critical strategy for pathway optimization due to its role in flux expansion. While both IUP and IPA pathways show significant potential, their industrial implementation requires further mechanistic optimization, particularly in phosphokinase screening and pathway regulation. These heterologous pathways are expected to be employed in the biosynthesis of sesquiterpenols to enhance production yields.

Downregulating the activity of metabolic branches can reduce metabolic flux loss, thereby enhancing the efficiency of the target product synthesis (Figure 4a, Table 2). The biosynthesis pathways of squalene and ergosterol are major competing pathways for sesquiterpenol production in yeast. Farnesyl diphosphate (FPP) is predominantly utilized for squalene synthesis through the high expression of squalene synthase (ERG9) rather than for sesquiterpenol synthases, diverting metabolic flux toward these competing pathways [114]. Downregulating squalene biosynthesis enables redirection of FPP precursors toward sesquiterpenol synthesis. However, completely knocking out the squalene and ergosterol pathways remains unfeasible due to their essential roles in cellular physiology. Current suppression strategies often involve promoter engineering, such as replacing the native ERG9 promoter with regulated ones, like PMET3 (methionine-repressible) or PHXT1 (glucose-responsive). The PHXT1 promoter provides better dynamic control [115]. Junqi Guo et al. achieved a record farnesol titer of 393.13 mg/L under flask culture conditions in *S. cerevisiae* by implementing PHXT1-driven ERG9 regulation [55]. Additionally, Jiang et al. introduced the MrBBS gene for (-)-α-bisabolol biosynthesis into the *S. cerevisiae* genome. Initially, no (-)-α-bisabolol was detected. To resolve this, the native promoter of the squalene synthase gene ERG9 was replaced with the HXT1 promoter. This strategy led to (-)-α-bisabolol production reaching 75.83 mg/L under flask fermentation conditions [73]. These findings demonstrate that downregulating ERG9 expression effectively enhances sesquiterpenol biosynthesis by redirecting metabolic flux toward target pathways. Complementary approaches involve blocking sesquiterpenol degradation by knocking out *DPP1* and *LPP1*, thereby conserving FPP flux for target compounds. Liu et al. demonstrated this by disrupting three extraneous pathways, including the farnesol synthase genes *DPP1* and *LPP1*, which led to physiological adaptations and 1.63 g/L patchoulol production [77]. Additionally, research has shown that the alkaline phosphatase *PhoA* from *E. coli* can hydrolyze geranylgeranyl pyrophosphate (GGPP) to produce geraniol. Drawing on this finding, Lu et al. hypothesized that the endogenous phospholipase in *S. marcescens* might recognize farnesyl pyrophosphate (FPP) and catalyze its hydrolysis to produce farnesol. To test this hypothesis, they used the CRISPR-Cas9 system to knockout the phospholipase gene *phoA* in *S. marcescens*, resulting in a 70% reduction in farnesol titers and an increase in (-)-α-bisabolol production to 3.21 g/L [5]. These findings establish that attenuating branch pathways is an effective strategy for enhancing metabolic flux partitioning toward desired.

While these strategies significantly enhance sesquiterpenol production, excessive gene overexpression might impose metabolic burdens that affect cellular fitness. Emerging solutions include AI/ML-assisted dynamic regulation systems [116]. These systems integrate multi-omics data to achieve orthogonal control between growth and product synthesis, enabling real-time pathway optimization in response to environmental and metabolic fluctuations.

#### 4.2.2. The Enhancement of the Central Carbon Flux

Within complex metabolic networks, multiple biosynthetic pathways coexist, often depleting central carbon flux and leading to by-product accumulation. Acetyl-CoA, a key metabolic intermediate in cellular processes, is essential for producing biotransformation-derived bio-based chemicals, and serves as a central node in microbial carbon metabolism. In yeasts, where sesquiterpenol biosynthesis primarily relies on the cytosolic acetyl-CoA pool as the starting substrate for the MVA pathway, enhancing acetyl-CoA availability is a strategic approach to improve production (Figure 5b, Table 2). Acetyl-CoA primarily originates from two sources: mitochondrial generation via ATP-citrate lyase in the TCA cycle and the cytoplasmic pyruvate dehydrogenase (PDH) bypass pathway [117]. Current enhancement strategies mainly focus on overexpressing ATP-citrate lyase (ACL) and optimizing the PDH bypass. In squalene-producing systems, heterologous expression of *Salmonella enterica* acetyl-CoA synthase (acs*) and endogenous ylACL1 increased cytosolic acetyl-CoA levels by over 50% [118]. Tao et al. used a strong promoter to overexpress ADH2 and ACS2, which encode acetyl-CoA synthase in *S. cerevisiae*. This enhanced the PDH bypass and endogenous acetyl-CoA flux, boosting patchoulol production by 1.34-fold to 151.27 ± 4.77 mg/L [76]. The peroxisomal β-oxidation pathway also significantly contributes to the acetyl-CoA pool. Ma et al. optimized this pathway in α-bisabolol-producing strains by balancing acetyl-CoA partitioning between endogenous lipid metabolism and heterologous biosynthesis, achieving 364.23 mg/L α-bisabolol production [70]. Recent advancements include the development of synthetic acetyl-CoA (SACA) pathways. These artificial biosynthetic pathways enable acetyl-CoA synthesis from formaldehyde through three enzymatic steps. This is the first engineered acetyl-CoA pathway and the shortest known biosynthetic route to acetyl-CoA, offering promising applications for sesquiterpenol bioproduction [119]. Collectively, these efforts demonstrate that strategically enhancing acetyl-CoA supply is an effective approach to improving sesquiterpenol biosynthesis efficiency.

Enhancing acetyl-CoA supply can boost central carbon flux and target compound biosynthesis. However, static methods often disrupt cellular homeostasis and fail to achieve central carbon flux distribution between cell growth and product synthesis. Recent advances in synthetic biology are driving the development of increasingly sophisticated dynamic regulation strategies [120]. Emerging solutions integrate biosensors [121] and genetic circuits for real-time flux control, coupled with genome-scale metabolic models (GEMs) [122] for in silico pathway optimization through computational simulations and mathematical modeling. These intelligent systems dynamically coordinate cellular growth phases with production stages, facilitating adaptive resource allocation. This technological convergence between dynamic regulation and predictive modeling is poised to revolutionize terpenoid biomanufacturing. It offers unparalleled metabolic engineering precision and enhances the fundamental capabilities of synthetic biology platforms.

### 4.3. Subcellular Organelle Engineering

The biosynthesis of terpenoids in model microbial chassis or plant cell factories encounters inherent limitations, including cofactor imbalance, substrate competition, product toxicity, and multi-enzymatic pathway complexity [123]. Cellular compartmentalization has emerged as a strategic solution to these challenges through subcellular spatial organization (Figure 5c, Table 2). Most cellular metabolic processes are naturally compartmentalized, with distinct organelles providing specialized microenvironments. These organelles optimize biochemical reactions by spatially regulating enzymatic cascades and metabolite channeling [124]. Eukaryotic organelles enclosed by phospholipid bilayers offer unique biochemical potentials through compartment-specific resources, such as hydrogen gradients, cofactor pools, and enzymatic systems [125]. In yeast, the most frequently employed compartments for sesquiterpenol synthesis are peroxisomes and mitochondria. Peroxisomes contain acetyl-CoA generated from fatty acid β-oxidation, supporting mevalonate (MVA) pathway operation. Liu et al. introduced the trans-nerolidol synthesis pathway genes into peroxisomes in two steps using an enhanced peroxisome targeting sequence (ePTS1). The resultant strain achieved a trans-nerolidol yield of 930 mg/L, which was 31% higher than that of the previous strain [66]. Similarly, Zhao et al. introduced the α-bisabolol synthesis pathway into *Y. lipolytica* peroxisomes, achieving a yield of 140.3 mg/L α-bisabolol [72]. Mitochondria provide high levels of ATP/NAD(P)H, and serve as reservoirs of acetyl-CoA. Luo et al. increased patchoulol production 1.7-fold (to 109 mg/L) in *P. pastoris* by incorporating a mitochondrial targeting signal peptide into the biosynthetic pathway [79]. Tao et al. integrated the MTS-PT gene into the genome of *S. cerevisiae* to compare patchoulol production in the cytoplasm and mitochondria. The engineered strain produced 1.79 ± 0.06 mg/L patchoulol, which was 2.71-fold higher than the reference strain [78]. These results indicate that mitochondria-engineered yeast cells have significant potential for enhancing patchoulol biosynthesis. In prokaryotic systems, protein-based structures such as bacterial microcompartments (BMCs) and membraneless organelles (MLOs) are employed for metabolic regulation [126]. BMCs facilitate substrate channeling and cofactor recycling, making them valuable for encapsulating terpenoid pathways. MLOs, which are liquid droplets formed through liquid–liquid phase separation, enable the dynamic spatial organization of enzymatic complexes. Both BMCs and MLOs hold promise for improving sesquiterpenol production yields in *E. coli* -based biosynthesis.

These compartmentalization strategies are effective across various microbial hosts. Utilizing synthetic biology tools, such as targeting peptides and scaffold proteins in combination with native organelle biochemistry, establishes a robust framework. This approach helps overcome bottlenecks in terpenoid biosynthesis, particularly in sesquiterpenol production. Future developments in programmable compartment engineering and dynamic pathway regulation are expected to enhance spatial control of metabolic flux.

### 4.4. Toxicity Tolerance of Terpenoids

Microbial cell factories encounter complex physiological and non-physiological challenges during terpenoid biosynthesis, such as high temperatures, high ethanol concentrations, high osmolarity, high viscosity, and substrate toxicity [127]. Collectively, these extracellular stressors impair normal protein activity and compromise membrane integrity. These issues trigger excessive reactive oxygen species (ROS) generation, primarily through protein denaturation in the electron transport chain. They also cause organelle dysfunction and metabolic disturbances [128]. Strengthening chassis resilience against terpene toxicity is crucial for enhancing sesquiterpenol yields (Figure 5d, Table 2). Antioxidant system engineering is a widely adopted strategic approach to mitigate these effects. Overexpressing antioxidant enzymes, such as catalase and superoxide dismutase, can reduce intracellular reactive oxygen species (ROS) levels. Liu et al. achieved a 4.1-fold increase in patchoulol production through the overexpression of cytosolic CTT1 and peroxisomal CTA1 [77]. In a previous study, Sun et al. demonstrated that the unfolded protein response (UPR) is triggered in yeast strains producing nerolidol, primarily due to endoplasmic reticulum stress. To address this issue, they introduced the human anti-apoptotic protein Bcl-2 into the yeast, which significantly enhanced nerolidol synthesis [129]. This research offers an effective, straightforward strategy for boosting sesquiterpenol production in engineered yeast. Enhancing the supply of NADPH can also strengthen cellular redox balance, thereby bolstering chassis resilience. Xu et al. increased farnesol production in *Rhodobacter sphaeroides* by overexpressing glucose-6-phosphate dehydrogenase (zwf) and 6-phosphogluconate dehydrogenase (gnd), thereby increasing NADPH levels [130]. Modulating efflux pumps to enhance cellular export and tolerance shows great potential for boosting the production of heterologous terpenoids in *E. coli* [131]. Zhou et al. explored efflux pumps to enhance patchoulol production by evaluating various transmembrane transporters. They overexpressed and modulated proteins such as TolC, ABC transporters (MacAB, MsbA), LptABCDFG, and YadGH in *E. coli*. To mitigate cytotoxicity caused by overexpression of membrane proteins, they used a subtilin-inducible promoter to control the expression of efflux transporters. The results showed that overexpressing MacAB and MsbA significantly increased patchoulol production by optimizing hydrophobic compound extrusion, reducing intracellular metabolite accumulation, and maintaining cellular viability [74]. Another approach to mitigating the toxicity of terpenoids is bioderivatization, which transforms the target terpenoid molecule into a less toxic derivative. This derivative can revert to the original terpenoid, when required, following the synthesis process. Terpene glycosylation can reduce cytotoxicity while maintaining recoverable products. Nerolidol glucoside exhibits superior ROS scavenging versus free nerolidol, thereby mitigating oxidative damage in plants [132]. Heterologous expression of glucosyltransferase ugt91q2 in *S. cerevisiae* or *E. coli* enables in situ nerolidol glycosylation, thereby enhancing production capacity [133].

These strategies establish toxicity tolerance as a cornerstone for industrial sesquiterpenol production. Future directions include CRISPR-engineered dynamic regulation of efflux systems and machine learning-guided design of glycosylation enzymes for tailored bioderivatization.

## 5. Conclusions

Sesquiterpenols, with their structural diversity and significant pharmacological potential, represent a key class of bioactive compounds. Compared to conventional plant extraction and chemical synthesis methods, engineering microbial cell factories for sesquiterpenol production offers a promising alternative. Despite notable advances in microbial engineering for sesquiterpenol production, several challenges hinder large-scale industrial applications. First, metabolic pathway regulation requires more comprehensive understanding. While the native MVA and MEP pathways are tightly regulated, their control networks, particularly in the MEP pathway, remain incompletely understood. This complexity limits the efficient redirection of metabolic flux toward target products. Therefore, deeper insights into these regulatory mechanisms are essential for precise control of the MVA and MEP pathways, providing a theoretical basis for enhancing sesquiterpenol yield and efficacy. Second, the structure–function relationships of sesquiterpenol synthases warrant immediate investigation. While protein engineering can enhance the catalytic efficiency of sesquiterpenol synthases, our understanding of their tertiary structures and catalytic mechanisms remains limited. Elucidating the spatial structures and comprehending the catalytic mechanisms of these synthases is crucial for advancing sesquiterpenol metabolic engineering. Third, high-throughput screening platforms are urgently needed. A significant limitation in terpenoid metabolic engineering is the absence of high-throughput screening methods, which hampers the efficiency of metabolic engineering strategies. New screening technologies, such as fluorescence-based or colorimetric assays, could accelerate the identification of high-producing strains and the optimization of biosynthetic pathways. Fourth, carbon conversion efficiency must be significantly improved. The existence of competing metabolic pathways often results in relatively low carbon conversion efficiency for sesquiterpenols. These pathways consume valuable carbon sources and release CO_2_ during decarboxylation reactions, thereby reducing overall production efficiency. Thus, enhancing carbon source conversion efficiency through pathway design and metabolic engineering is imperative for cost-effective sesquiterpenol production. Finally, by-product formation during production requires mitigation. In sesquiterpenol production, by-product formation not only reduces the yield of the target product but increases downstream purification costs. Therefore, employing metabolic engineering strategies to minimize or eliminate these by-products is a vital approach to improving production efficiency and reducing costs.

In summary, the microbial production of sesquiterpenols faces multiple challenges, ranging from elucidating the fundamental mechanisms of metabolic pathway regulation to addressing practical aspects such as improving enzyme catalytic efficiency, developing high-throughput screening technologies, and optimizing carbon source conversion efficiency. Overcoming these challenges requires interdisciplinary research efforts, including close collaboration across fields such as molecular biology, biochemistry, computational biology, and chemical engineering. With the continued advancements in synthetic biology and systems biology, future research in these areas will provide new strategies and tools for the efficient production of sesquiterpenols.

## Figures and Tables

**Table 1:** Functions of different sesquiterpenols.

Classification	Sesquiterpenol	Functions and Applications	References
acyclic	nerolidol	antimicrobial, anti-biofilm, antioxidant, antiparasitic, transdermal permeation-enhancing, antinociceptive, and anticancer properties	[11,12,13,14,15,16,17]
	farnesol	essential oils, intercellular signaling, quorum sensing modulation, apoptosis regulation	[19]
monocyclic	(-)-α-bisabolol	ulcer healing, gallstone dissolution, analgesia, anti-inflammatory, bacteriostasis, anti-genotoxic, melanin inhibition	[9,20]
	elemol	fragrance formulation, personal care products, laundry detergents, household commodities	[21]
bicyclic	eudesmol	fragrance fixative, antimicrobial, insecticidal properties	[22]
	τ-cadinol	pest-repellent, nutritional substrate, membrane stabilizer, energy metabolism modulator, smooth muscle relaxation, antidiarrheal effects, calcium channel antagonism, phyto germination regulation, acaricidal action, insect repellency	[23,24,25,26]
polycyclic	patchoulol	woody aroma, cosmetics and household products, anti-anginal activity, arrhythmia suppression, antihypertensive effects	[27]
	α-santalol	antitumor, antimicrobial, antibacterial prophylaxis, virucidal activity, antioxidant properties	[28,29,30,31]
	cedrol	oriental fragrance formulations	[32,33,34]
	curcumol	anticancer, antimicrobial, antifungal, antiviral, anti-inflammatory	[35]

**Table 2:** Summary of strategies for the sesquiterpenols heterologous production.

Classification	Sesquiterpenol	Host	STRATEGIES	Scales	Titers (mg/L)	Reference
Acyclic	Z,Z- farnesol	*Escherichia coli*	tested five Z, Z-farnesyl diphosphate (Z, Z-FPP) synthases; screened thirteen phosphatases; site-directed mutagenesis of cis-prenyltransferase	flask	572.13	[6]
	farnesol	*Saccharomyces cerevisiae*	overexpressed tHMGR1 and ERG20; replacement of the natural ERG9 promoter with the hxt1 promoter; overexpressed of endogenous phosphatase PAH1	flask	393.13	[55]
	farnesol	*Saccharomyces cerevisiae*	replaced the natural ERG9 promoter with the tunable MET3 promoter	flask	20.2	[60]
	farnesol	*Saccharomyces cerevisiae*	overexpressed tHMGR1	bioreactor	145.7	[61]
	farnesol	*Escherichia coli*	overexpressed ispA; introduced the MVA pathway	flask	135.5	[62]
	nerolidol	*Saccharomyces cerevisiae*	fused of nerolidol synthase to farnesyl diphosphate synthase	flask	4200	[56]
	nerolidol	*Saccharomyces cerevisiae*	depleted hexokinase-2 to lifts glucose repression; depleted acetyl-CoA carboxylase (Acc1p)	flask	3500	[63]
	nerolidol	*Saccharomyces cerevisiae*	engineered a eukaryote-like tetracycline-mediated circuit to minimize metabolic burden during strain development and maintenance	flask	2540	[64]
	trans- nerolidol	*Saccharomyces cerevisiae*	overexpressed the mevalonate pathway; knockout of GAL80 gene; replaced the natural ERG9 promoter with the tunable HXT1 promoter	bioreactor	7010	[65]
	nerolidol	*Yarrowia lipolytica*	used homology modeling and docking studies to design the FaNES1^G498Q^ mutant of nerolidol synthase; enhanced the expression of an endogenous carnitine acetyltransferase (CAT2); introduced the nerolidol biosynthesis pathway into the peroxisomal	bioreactor	11,100	[66]
	nerolidol	*Escherichia coli*	overexpressed nerolidol synthase; fusion nerolidol synthase and ispA	flask	0.016	[67]
	nerolidol	*Saccharomyces cerevisiae*	overexpressed the MVA pathway synthase; engineered CLN2^PEST^ sequence-dependent degradation of ER-associated protein with the Erg9p signal peptide	flask	150	[68]
Monocyclic	α-bisabolol	*Pichia pastoris*	optimized the mevalonate pathway; peroxisomal compartmentalization; overexpressed the limiting enzyme EfmvaE	flask	1100	[69]
	α-bisabolol	*Yarrowia lipolytica*	overexpressed tHMG1 and ERG20; truncated the native promoter of the squalene synthase gene SQS1; increased the copy number of the bisabolenol synthase gene MrBBS;enhanced the β-oxidation pathway	flask	364.23	[70]
	α-bisabolol	*Yarrowia lipolytica*	used YALI clone NHEJ system to overexpress the mevalonate pathway;	bioreactor	4400	[71]
	α-bisabolol	*Yarrowia lipolytica*	introduced the α-bisabolene biosynthesis pathway into the peroxisomal; efflux pump-mediated product export; optimized the gene copy number of rate-limiting enzymes; balanced the distribution of the common precursor acetyl-CoA between natural lipids	bioreactor	15,500	[72]
	α-bisabolol	*Saccharomyces cerevisiae*	used the low-transcription-level HXT1 promoter to replace the initial promoter of ERG9; fusion expression of ERG20 and MrBBS; knockout of DPP1; overexpressed ERG20 and ADH2; overexpressed global activator of MVA pathway Upc2^G888A^; knockout of the ROX1 and Ypl062w	bioreactor	7020	[73]
	α-bisabolol	*Serratia marcescens*	introduce heterology MVA pathway; knockout of PhoA; co-expression DnaK/J	bioreactor	30,200	[5]
Bicyclic	τ-cadinol	*Escherichia coli*	overexpressed of IDI; two-phase fermentation	flask	133.5	[26]
	τ-cadinol	*Escherichia coli*	knock out the cAMP synthesis pathway; constructed a glycolysis-control device mediated by pyruvate sensing; dynamic regulated the glycolysis	bioreactor	15,200	[26]
Polycyclic	patchoulol	*Escherichia coli*	overexpressed the exogenous mevalonate pathway; overexpressed a patchoulol synthase; optimized the pH and temperature of culture broth	bioreactor	40.2	[58]
	patchoulol	*Escherichia coli*	semi-rational mutant Patchoulol synthase (PTS); fused of PTS with FPP synthase; deleted the competitive routes for acetate, lactate, ethanol, and succinate synthesis; enhanced the expression of efflux transporters	bioreactor	970.1	[74]
	patchoulol	*Escherichia coli*	knocked out the cAMP synthesis pathway to alleviate glucose repression; constructed a glycolysis-control device mediated by pyruvate sensing; dynamic regulated the glycolysis	bioreactor	1675.1	[42]
	patchoulol	*Saccharomyces cerevisiae*	optimized the expression of eight genes in the MVA pathway: integrated multiple copies of the patchoulol synthase gene (LibPTS)	flask	42.1	[75]
	patchoulol	*Saccharomyces cerevisiae*	overexpressed the mevalonate pathway; integrated multiple copies of the patchoulol synthase gene; knocked out the farnesol synthase genes DPP1 and LPP1; replaced the natural ERG9 promoter with the tunable ERG1 promoter; enhanced the supply of acetyl-CoA by overexpressing ADH2 and knocking out the transcription factors YPL062W, YNR063W, and YJL064W	flask	195.96	[76]
	patchoulol	*Saccharomyces cerevisiae*	overexpressed tHMGR1; fusion of FPP synthase ERG20 and PTS; replaced the natural ERG9 promoter with the tunable HXT1 promoter; enhanced the supply of acetyl-CoA by knocking out the transcription factors YPL062W, YNR063W, and YJL064W; overexpressed the cytoplasmic catalase CTT1 and the peroxisomal catalase CTA1 to reduce the levels of reactive oxygen species (ROS);constructing the PTS mutant PTS^T404S^	flask	141.5	[77]
	patchoulol	*Saccharomyces cerevisiae*	fusion of farnesyl pyrophosphate (FPP) synthase and patchoulol synthase to increase the utilization of FPP precursors; enhanced the expression of rate-limiting genes in the mevalonate pathway; squalene synthase was weakened by the glucose-inducible promoter of HXT1 to reduce the metabolic flux from FPP to ergosterol; inhibited the biosynthesis of farnesol, thereby reducing the consumption of FPP	flask	466.8	[44]
	patchoulol	*Saccharomyces cerevisiae*	fusion of farnesyl pyrophosphate (FPP) synthase and patchoulol synthase; introducing the patchoulol biosynthesis pathway into the mitochondria of *Saccharomyces cerevisiae*	flask	19.24	[78]
	patchoulol	*Pichia pastoris*	dual cytoplasmic–mitochondrial engineering; introducing the patchoulol biosynthesis pathway into the mitochondria	flask	109	[79]
	patchoulol	*Pichia pastoris*	fusion of farnesyl pyrophosphate (FPP) synthase and patchoulol synthase; increased precursor supply; added auxiliary carbon source	flask	149.64	[80]
	patchoulol	*Yarrowia lipolytica*	downregulated squalene synthesis based on Cu^2+^-repressible promoter; expanded the mevalonate precursor pool; fusion of farnesyl pyrophosphate (FPP) synthase and patchoulol synthase	flask	235	[81]
	santalol	*Saccharomyces cerevisiae*	downregulated ERG9 gene; truncated the N-terminal 46 amino acid of ATR1	flask	68.8	[59]
	santalol	*Saccharomyces cerevisiae*	replaced the natural ERG9 promoter with the tunable HXT1 promoter; overexpressed santalol synthesis pathway; overexpressed GAL4 (a transcriptional activator of *GAL* promotors) and PGM2 (a yeast phosphoglucomutase)	bioreactor	1300	[82]

**Table 3:** Summary of identified crucial genes involved in sesquiterpenols production.

Crucial Genes	Sources	Involved in Biosynthesis of Which Sesquiterpenols	Validation Strategies	Reference
farnesyl diphosphate synthase; α-bisabolol synthase	*Matricaria recutita* L.	α-bisabolol	purification from tissue; in vitro biochemical assays; transcriptomics;	[87]
α-bisabolol synthase	*Lippia dulcis*	α-bisabolol	transcriptomics; biochemical assays; heterologous expression in *Saccharomyces cerevisiae*	[88]
nerolidol synthase	*Actinidia chinensis*	nerolidol	purification from tissue; transcriptomics; in vitro biochemical assays;	[83]
nerolidol synthase	*Celastrus angulatus*	nerolidol	purification from tissue; transcriptomics; in vitro biochemical assays; heterologous expression in *Saccharomyces cerevisiae*	[65]
nerolidol synthase	*Camellia sinensis*	nerolidol	transcriptomics; purification from tissue;	[84]
α-bisabolol synthase	*Sesamum indicum* L.	α-bisabolol	transcriptomics;	[89]
α-bisabolol synthase	*Arabidopsis thaliana*	α-bisabolol	genomics;	[90]
nerolidol synthase	*Cannabis sativa*	nerolidol	genomics;	[85]
nerolidol synthase	*peanut*	nerolidol	global transcriptome analysis	[86]
farnesol synthase	*Cryphonectria parasitica*	farnesol	genomics;	[91]
elemol synthase	*potato*	elemol	purification from tissue; transcriptomics;	[92]
cytochrome P450 monooxygenase	*Santalum album*	α-santalol	transcriptomics;	[93]
eudesmol synthase	*Atractylodes lancea*	eudesmol	transcriptomics; genomics	[94]
τ-cadinol synthase	*Zea mays*	τ-cadinol	purification from tissue; in vitro biochemical assays;	[95]
τ-cadinol synthase	*Lavandula angustifolia*	τ-cadinol	transcriptomics	[96]
cedrol synthase	*Euphorbia fischeriana*	cedrol	transcriptomics; in vitro biochemical assays;	[97]

## Data Availability

Not applicable.
